# Effect of Reprocessed Micronized Acellular Dermal Matrix on Postoperative Dysphagia After Anterior Cervical Discectomy and Fusion: A Propensity Score-Matched Study

**DOI:** 10.3390/medicina62061163

**Published:** 2026-06-15

**Authors:** Dong Hun Kim, Jung-Woo Hur, Jae-Taek Hong

**Affiliations:** 1 Department of Neurosurgery, Bucheon St. Mary’s Hospital, College of Medicine, The Catholic University of Korea, Seoul 14647, Republic of Korea; 2 Department of Neurosurgery, Eunpyeong St. Mary’s Hospital, College of Medicine, The Catholic University of Korea, Seoul 03312, Republic of Korea

**Keywords:** acellular dermis, anterior cervical discectomy and fusion, deglutition disorders, tissue adhesions, postoperative complications, propensity score matching

## Abstract

*Background and Objectives*: Postoperative dysphagia following anterior cervical discectomy and fusion (ACDF) remains a common complication affecting patient quality of life. This study evaluated the safety and clinical efficacy of reprocessed micronized acellular dermal matrix (ADM) compared to conventional gel-type anti-adhesive agents in patients undergoing single-level ACDF. *Materials and Methods*: This retrospective propensity score-matched study included 108 patients (54 matched pairs) who underwent single-level ACDF between January 2021 and December 2025. The ADM group received CGDerm Matrix™ and the control group received Mediclore™. The primary outcome was postoperative swallowing function assessed by the Swallowing Impairment Score (SIS-6) at 3 months (pre-specified primary time point). Secondary outcomes included VAS, NDI, modified MacNab criteria, adhesion scores, prevertebral soft tissue swelling, and perioperative inflammatory markers. Bonferroni correction was applied for multiple comparisons (adjusted α = 0.0125). *Results*: The ADM group demonstrated significantly lower SIS-6 scores at 3 months (0.26 ± 0.16 vs. 0.68 ± 0.27, *p* = 0.01), which remained significant after Bonferroni correction. All other clinical and radiological outcomes were comparable between groups. No device-related complications occurred. *Conclusions*: ADM application in ACDF surgery appears safe and is associated with improved postoperative swallowing function at 3 months. However, the clinical significance of observed differences requires further investigation.

## 1. Introduction

Anterior cervical discectomy and fusion (ACDF) has become a standard surgical procedure for treating degenerative cervical disc disease, including disc herniation and symptomatic spinal stenosis [1,2]. The anterior plating system has significantly improved fusion rates and cervical alignment, contributing to favorable surgical outcomes [3]. However, this approach requires substantial esophageal retraction, which can lead to postoperative complications such as dysphagia, hoarseness, and decreased quality of life [4,5].

Postoperative dysphagia is one of the most common complications following ACDF surgery, with reported incidence rates ranging from 12.5% to 35.1% for persistent symptoms lasting more than three months [2,6]. Despite its high incidence, the etiology of postoperative dysphagia remains relatively unknown, its treatment is largely unexplored, and risk factors are widely debated [7,8]. Several potential causes have been suggested, including the thickness of the cervical plate, prevertebral soft tissue swelling, and significant esophageal retraction during surgery [9,10]. Various factors such as age, sex, body mass index, operative time, and number of surgical levels have been studied as possible risk factors with inconsistent findings across multiple studies [3,11].

Unwanted adhesion is considered a significant contributor to postoperative pain, dysphagia, and hoarseness [12,13]. Various anti-adhesive agents, including fibrinolytic agents, film/membrane barriers, and gel-type products, have been used to prevent adhesion formation [14,15]. However, no established method has been reported to demonstrate excellent effect in preventing postoperative adhesion [16].

Acellular dermal matrix (ADM) is a tissue-engineered scaffold that supports intrinsic self-healing through dynamic reciprocity with host tissues [17,18]. It retains natural biological components that can be repopulated by the patient’s cells, allowing the body to initiate its own tissue regeneration process. The processing of ADM removes viable cells and antigens, which minimizes inflammation or rejection at the surgical site [19,20]. ADM has been widely used for the past 20 years in surgical reconstruction, with well-demonstrated long-term safety and efficacy [21,22]. It provides a biomimetic surface for cell attachment, matches the elasticity and stiffness of dermis, and prevents hypertrophic scars or wound contractures [23].

Despite the extensive use of ADM in various surgical fields, there are no published data regarding its application in spine surgery. The purpose of this study was to evaluate the efficacy and safety of novel reprocessed micronized ADM on postoperative swallowing function in patients undergoing conventional ACDF surgery through a retrospective observational study from a single surgeon’s experience at a single center.

## 2. Materials and Methods

### 2.1. Study Design and Ethical Approval

This retrospective observational study was conducted at a single tertiary academic center between January 2021 and December 2025. This study was approved by the Institutional Review Board of Eunpyeong St. Mary’s Hospital, The Catholic University of Korea (IRB No. PC22RISI0047, approved on 19 February 2026) and conducted in accordance with the Declaration of Helsinki. Patient consent was waived due to the retrospective nature of this study, in accordance with the Bioethics and Safety Act of the Republic of Korea (Article 16, Paragraph 3). This study was reported following the Strengthening the Reporting of Observational Studies in Epidemiology (STROBE) guidelines (Supplementary Checklist S1).

### 2.2. Patient Selection

Inclusion criteria were: (1) adults aged 18–80 years; (2) symptomatic single-level cervical disc disease (C3–C7) requiring ACDF; (3) degenerative indications including disc herniation, spinal stenosis, or degenerative disc disease; and (4) minimum 3-month postoperative follow-up.

Exclusion criteria were: (1) non-degenerative pathologies including infection, tumor, trauma, ankylosing spondylitis, or rheumatoid arthritis; (2) previous cervical spine surgery; (3) pre-existing dysphagia or esophageal pathology; (4) multi-level surgery; and (5) incomplete medical records.

### 2.3. Group Allocation and Surgical Technique

Group allocation was based on the period of surgery and product availability. ADM (CGDerm Matrix™, CGBio, Seongnam, Republic of Korea) was introduced at our institution in June 2023. Patients operated before this date received the conventional gel-type anti-adhesive agent (Mediclore™, CGBio, Seongnam, Republic of Korea), while patients operated after this date received ADM. This allocation was determined by product availability rather than surgeon preference or patient selection. To minimize potential confounding from temporal trends, propensity score matching was performed to ensure baseline comparability between groups.

**Figure 1:**
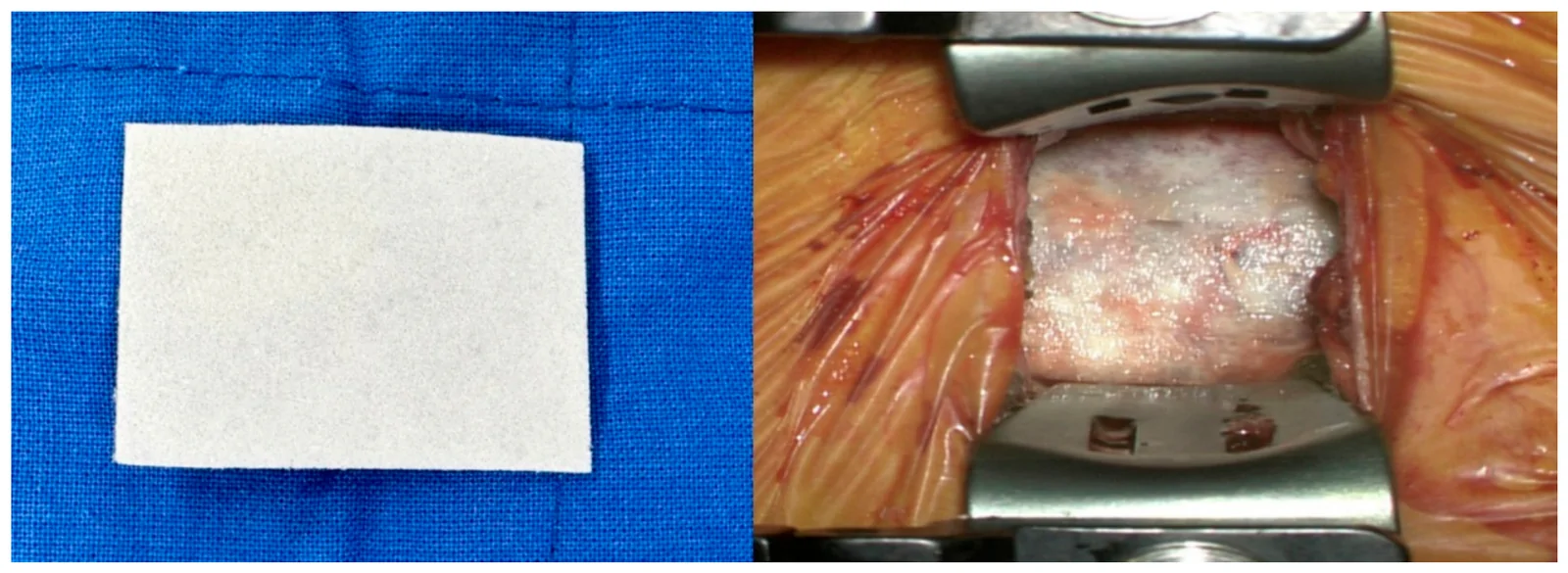
Representative intraoperative photographs showing reprocessed micronized ADM and saline-soaked ADM placed over the anterior cervical plate.

All surgeries were performed by a single surgeon using a standard Smith-Robinson approach [1]. Following discectomy and neural decompression, an allograft bone block (C-plus™, CGBio, Seongnam, Republic of Korea) was inserted into the disc space, and an anterior cervical plate system (Trisecure™, CGBio, Seongnam, Republic of Korea) was applied for fixation. Anti-adhesive agents were applied over the operation site between the anterior vertebral body/plate construct and the esophagus. The ADM group received 0.7 mm thickness reprocessed micronized acellular dermal matrix (Figure 1). The control group received a conventional sol/gel-type anti-adhesive agent.

### 2.4. Outcome Measures

Clinical outcome measures included postoperative patient-reported outcome measures (PROMs) such as VAS scores for neck and arm pain, Neck Disability Index (NDI), and modified MacNab criteria. Clinical and radiologic evaluations were performed preoperatively and at postoperative day 1, day 3, 1 month, and 3 months. Radiologic parameters were independently assessed twice by two spine surgeons with more than 10 years of clinical experience, and the mean values were used for analysis.

The primary outcome was postoperative swallowing function assessed by the Swallowing Impairment Score (SIS-6) at 3 months (pre-specified primary time point). The SIS-6 is a validated 6-item self-evaluation questionnaire originally developed by Lombardi et al. for assessing swallowing dysfunction following thyroidectomy [24]. Each item is scored from 0 (no impairment) to 4 (maximum impairment), with a total score ranging from 0 to 24 (higher scores indicating worse swallowing function). A Korean-translated version of the SIS-6 was used in this study. The questionnaire was translated according to standard forward-backward translation procedures; however, formal psychometric validation of the Korean version has not been performed. The complete SIS-6 questionnaire is provided in Supplementary Table S1. SIS-6 assessments at postoperative day 1, day 3, and 1 month were considered exploratory secondary time points.

Adhesion score was assessed using a questionnaire originally developed for thyroidectomy [25]. The questionnaire consists of 7 items divided into two domains: (1) subjective patient discomfort (3 items assessing difficulty in swallowing saliva, water, and solid food; scored 0–4 each); and (2) objective clinical assessment (4 items evaluating cervical appearance, symmetry at rest, symmetry with neck extension, and inflammatory reaction/scar formation; scored 0–4 each). Total score ranges from 0 (no adhesion-related symptoms) to 28 (maximum symptoms). The questionnaire was administered at each follow-up visit and scored by a clinical assessor blinded to group allocation.

Prevertebral soft tissue swelling was measured on lateral cervical radiographs obtained at each follow-up time point. Measurements were performed at the level of the operated disc space, from the anteroinferior corner of the vertebral body to the anterior border of the prevertebral soft tissue shadow (airway). All measurements were performed independently by two spine surgeons with more than 10 years of experience, and the mean values were used for analysis. Inter- and intra-observer reliability was assessed using intraclass correlation coefficients (ICC).

Serum inflammatory markers (CK, LDH, CRP, ESR) were measured to assess systemic biocompatibility and to evaluate whether ADM application induces any additional inflammatory or immunological response compared to conventional gel-type agents. Blood samples were collected preoperatively and at postoperative day 1, 2 weeks, 1 month, and 3 months. Analyses were performed at the hospital’s central laboratory using standardized protocols.

Postoperative MRI was performed in a subset of patients (*n* = 15 in ADM group, *n* = 12 in Non-ADM group) at 3 months postoperatively for evaluation of ADM integration and soft tissue changes. MRI was not performed in all patients due to routine clinical practice patterns. Imaging was reviewed by a neuroradiologist blinded to group allocation.

Adverse events were defined as any unfavorable medical occurrence during the follow-up period. Device-related complications were defined as adverse events potentially attributable to the anti-adhesive agent, including wound complications, infection, allergic reaction, or foreign body reaction. Readmission within 30 days and need for additional spine surgery were recorded.

### 2.5. Statistical Analysis

Retrospective medical record review identified 135 eligible patients. Propensity scores were calculated using logistic regression with the dependent variable being ADM use (ADM vs. non-ADM) and independent variables including age, sex, and operative level. These three covariates were selected based on their established association with post-ACDF dysphagia in the literature and consistent data availability across all patients. One-to-one propensity score matching yielded 54 matched pairs (108 patients total). Standardized mean differences (SMDs) before and after matching are reported in Supplementary Table S2; all post-matching SMDs were <0.1, indicating adequate covariate balance.

Normality of continuous variables was assessed using the Shapiro–Wilk test. Normally distributed continuous variables were compared using independent *t*-tests and reported as mean ± standard deviation. Non-normally distributed variables (intraoperative blood loss, postoperative surgical drainage) were analyzed using the Wilcoxon rank-sum test and reported as median (interquartile range). Categorical variables were analyzed using chi-square test or Fisher’s exact test.

Bonferroni correction was applied for the primary outcome (SIS-6) tested at four time points, with adjusted significance level α = 0.0125. The results were considered statistically significant at *p* < 0.05 for uncorrected analyses and *p* < 0.0125 for Bonferroni-corrected primary outcome analyses. Post hoc power analysis was performed using observed means and standard deviations for the primary endpoint. Statistical analyses were performed using SPSS Version 26.0 (IBM Corp., Armonk, NY, USA).

## 3. Results

### 3.1. Patient Demographics and Operative Data

A total of 108 patients were included in the final analysis after propensity score matching, with 54 patients in each group. There were no statistically significant differences in patient demographics between the two groups. The gender distribution was identical (male: 55.5%, female: 44.5% in both groups), and the mean age was 55.4 (±11.9) years in both groups (Table 1). Operative level distribution was: C3–4 (3.7%), C4–5 (20.4%), C5–6 (68.5%), and C6–7 (7.4%) in the ADM group; C3–4 (3.7%), C4–5 (18.5%), C5–6 (70.4%), and C6–7 (7.4%) in the Non-ADM group.

Preoperative symptoms were comparable between groups, with VAS for neck pain (7.9 ± 1.0 vs. 7.6 ± 1.5, *p* = 0.71), VAS for arm pain (8.8 ± 2.1 vs. 8.7 ± 2.2, *p* = 0.32), and NDI (28.7 ± 5.5 vs. 27.1 ± 9.7, *p* = 0.69). Symptom duration, body mass index, and comorbidities (hypertension, diabetes, osteoporosis, smoking) showed no significant differences. Intraoperative blood loss showed non-normal distribution and was reported as median (IQR): ADM group 50.0 mL (35.0–75.0) vs. Non-ADM group 52.5 mL (30.0–80.0), *p* = 0.85. Operating time, fluoroscopic time, length of hospital stay, and postoperative surgical drainage showed no significant differences between groups.

Complete follow-up data at 3 months (primary time point) were available for 52/54 (96.3%) patients in the ADM group and 53/54 (98.1%) patients in the Non-ADM group. At 1 month, follow-up was complete for 54/54 (100%) in both groups. Mean follow-up duration was 8.8 ± 3.8 months (ADM) and 7.8 ± 3.2 months (Non-ADM), *p* = 0.14.

### 3.2. Clinical Outcomes

**Figure 2:**
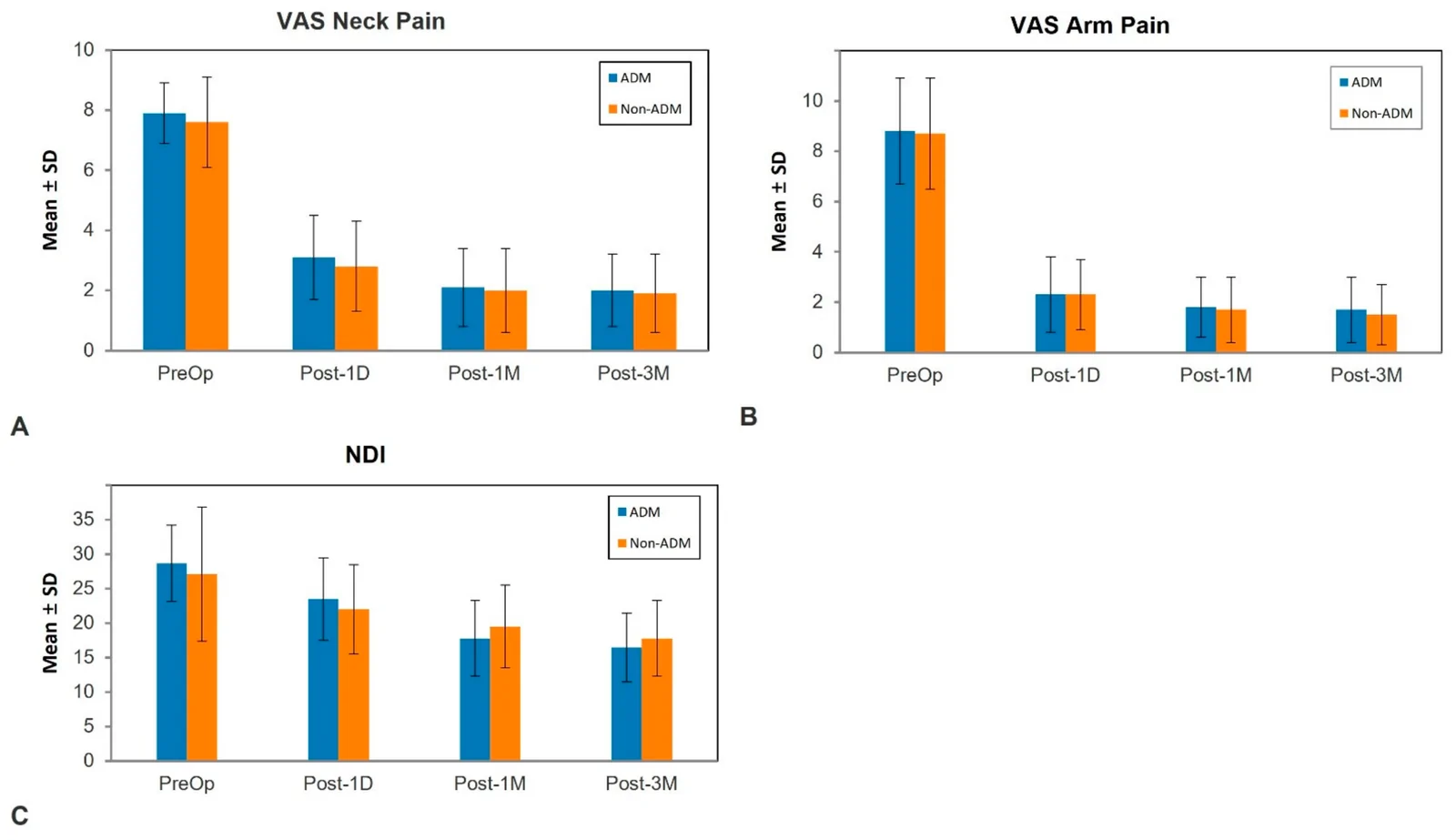
Comparison of clinical outcomes between the ADM and non-ADM groups over time. (**A**) VAS neck pain scores at preoperative and postoperative time points. (**B**) VAS arm pain scores at preoperative and postoperative time points. (**C**) NDI scores at preoperative and postoperative time points. Both groups showed significant improvement with no significant between-group differences. Error bars represent standard deviation. ADM, acellular dermal matrix; VAS, visual analogue scale; NDI, neck disability index; PreOp, preoperative; Post-1D, postoperative day 1; Post-1M, postoperative 1 month; Post-3M, postoperative 3 months.

Postoperative clinical outcomes improved significantly in both groups (Table 2). Neck pain VAS decreased from baseline (ADM: 7.9 ± 1.0; non-ADM: 7.6 ± 1.5) to 2.0 ± 1.2 and 1.8 ± 1.3 at 3 months. No significant differences were identified between groups at any time point (Figure 2A). Arm pain VAS also declined markedly with no between-group differences (Figure 2B). NDI scores decreased from 28.7 ± 5.5 and 27.1 ± 9.7 at baseline to 15.9 ± 5.0 and 17.8 ± 5.5 at 3 months, without significant between-group differences (Figure 2C). According to modified MacNab criteria, satisfactory results (excellent or good) were achieved in 51 patients (94.4%) in the ADM group compared to 48 patients (88.9%) in the control group (*p* = 0.43; Table 3).

### 3.3. Primary Outcome: Swallowing Function (Sis-6)

Swallowing function outcomes are summarized in Table 4. At the pre-specified primary time point (3 months), the ADM group demonstrated significantly lower SIS-6 scores compared to the Non-ADM group (0.26 ± 0.16 vs. 0.68 ± 0.27, *p* = 0.01), which remained statistically significant after Bonferroni correction (adjusted α = 0.0125).

In exploratory analyses at other time points, SIS-6 scores were comparable between groups at postoperative day 1 (ADM: 2.49 ± 1.41 vs. Non-ADM: 2.67 ± 1.44, *p* = 0.60) and day 3 (ADM: 1.75 ± 0.59 vs. Non-ADM: 1.93 ± 0.56, *p* = 0.20). At 1 month, the ADM group showed lower scores (1.09 ± 0.58 vs. 1.76 ± 0.53, uncorrected *p* = 0.03; however, this difference was not statistically significant after Bonferroni correction).

Post hoc power analysis using observed means and standard deviations at 3 months (ADM: 0.26 ± 0.16; Non-ADM: 0.68 ± 0.27), with α = 0.05 and *n* = 54 per group, yielded an achieved power of 99.8% for detecting the observed difference.

### 3.4. Secondary Outcomes: Adhesion Score and Soft Tissue Swelling

Total adhesion scores showed no statistically significant differences between groups at any postoperative time point (Table 5). Both groups showed improvement from immediate postoperative values to later time points. Mean prevertebral soft tissue swelling also showed no significant differences between groups (Table 4). The intra- and inter-observer reliability for measurement of prevertebral soft tissue swelling was excellent at all postoperative time points, with ICC values consistently greater than 0.88 (Table 6).

### 3.5. Perioperative Inflammatory Markers

**Figure 3:**
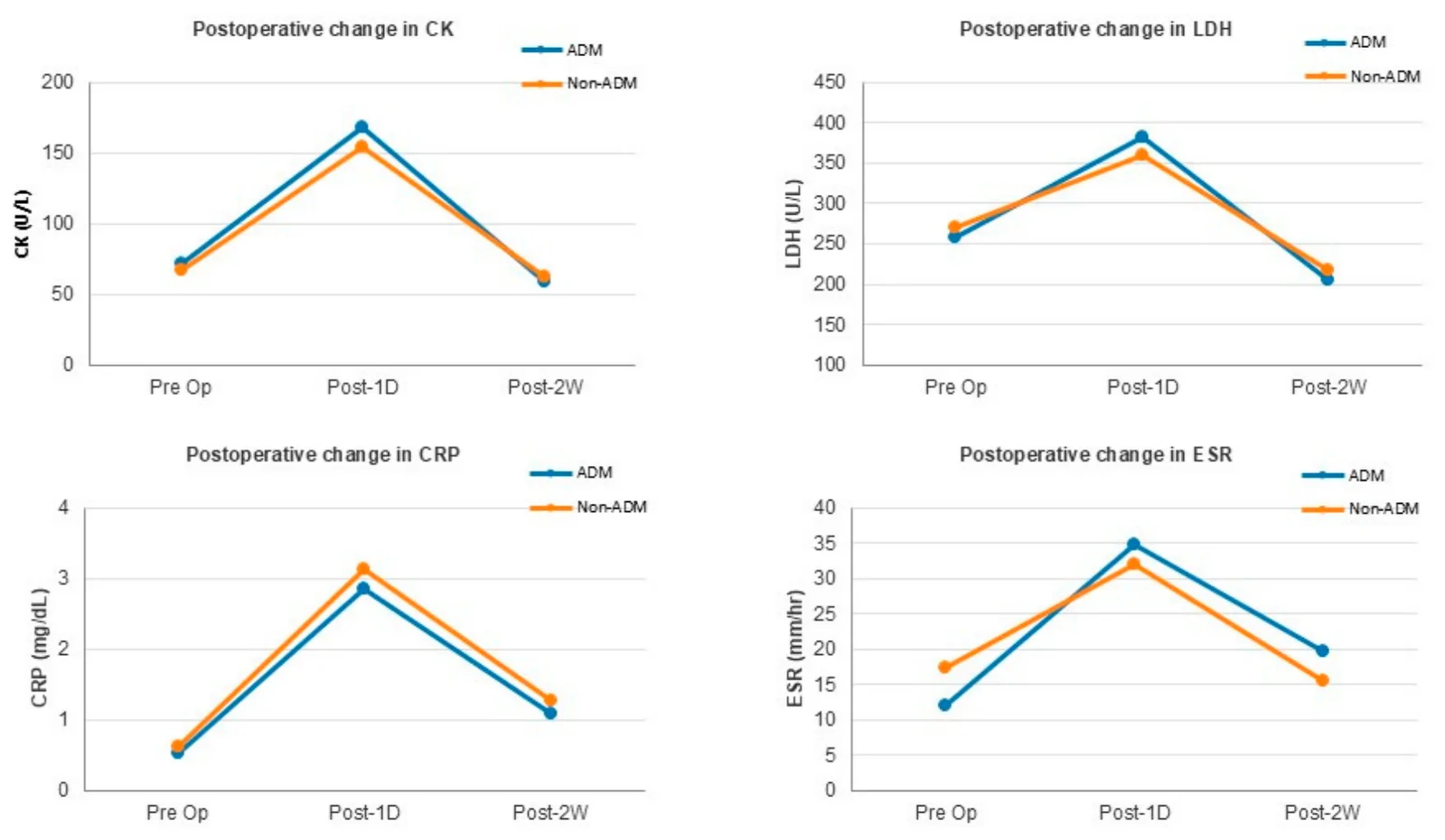
Comparison of serum inflammatory markers including CK, LDH, CRP, and ESR between ADM and Non-ADM groups at preoperative and postoperative time points. Both groups demonstrated typical postoperative elevation patterns with peak values on postoperative day 1, followed by gradual normalization. No significant differences were observed between groups at any time point. Error bars represent standard deviation. ADM, acellular dermal matrix; CK, creatine kinase; LDH, lactate dehydrogenase; CRP, C-reactive protein; ESR, erythrocyte sedimentation rate; PreOp, preoperative; Post-1D, postoperative day 1; Post-2W, postoperative 2 weeks.

Serum inflammatory markers demonstrated typical postoperative elevation patterns in both groups, with peak values on postoperative day 1, followed by gradual normalization (Figure 3, Table 7). No statistically significant differences were observed between groups at any time point.

### 3.6. Radiologic Findings and Safety Outcomes

**Figure 4:**
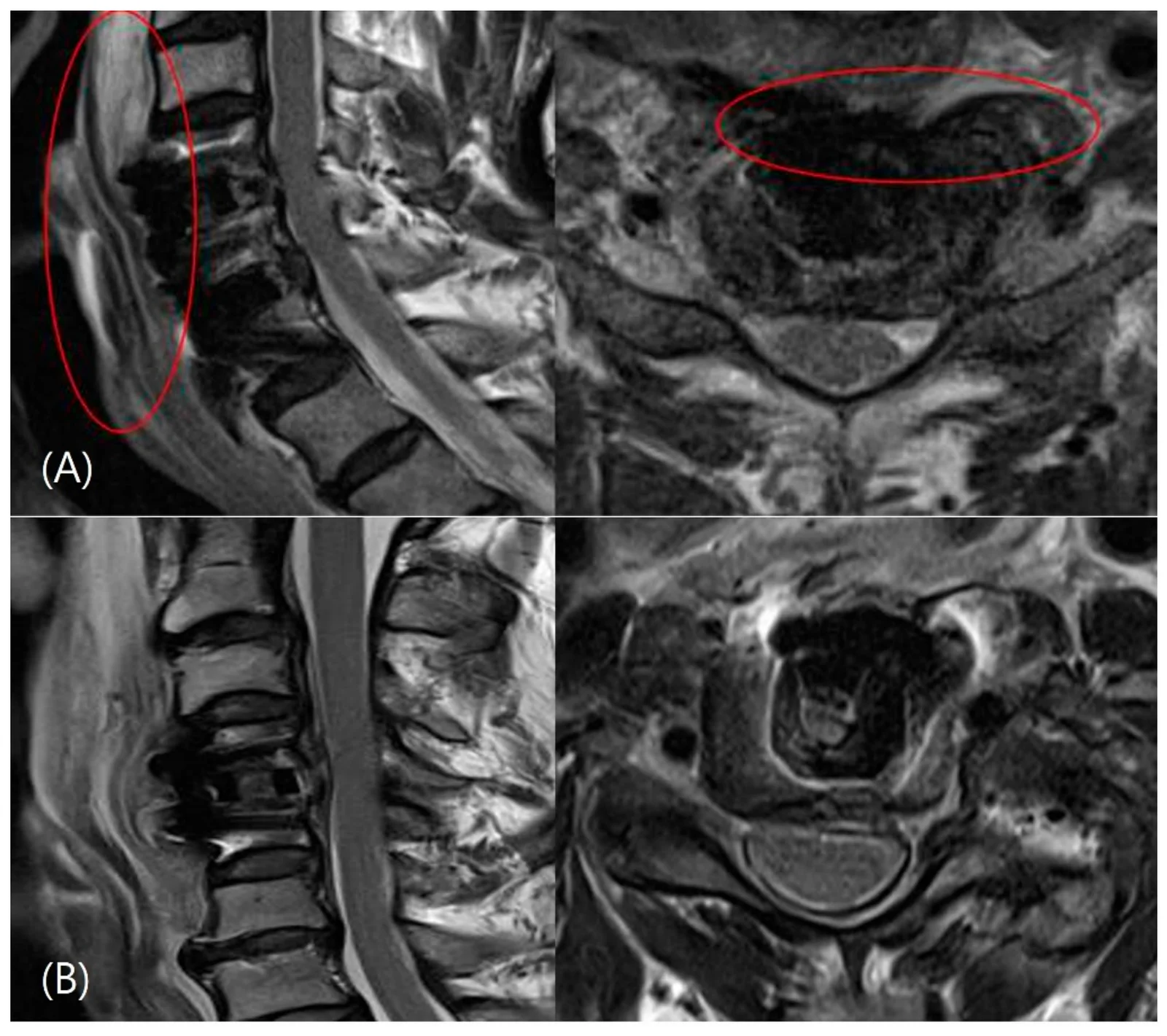
(**A**) Postoperative MRI images showing a thick intermediate-signal layer overlying the anterior cervical plate in the ADM group (red circles). This layer was interposed between the esophagus and the anterior instrumentation, consistent with the presence of the acellular dermal matrix material. ADM, acellular dermal matrix; MRI, magnetic resonance imaging. (**B**) Representative MRI from the Non-ADM group is shown for comparison.

Postoperative MRI demonstrated a thick intermediate-signal layer overlying the anterior cervical plate in patients receiving ADM, consistent with the presence of the matrix material (Figure 4). This layer was interposed between the esophagus and the anterior instrumentation. No device-related complications were observed in either group. There were no 30-day readmissions or additional spine surgeries required during the follow-up period.

## 4. Discussion

This study suggests that the use of novel reprocessed micronized ADM in ACDF surgery is safe and may be associated with improved recovery from postoperative swallowing dysfunction compared to conventional gel-type anti-adhesive agents. While the ADM group demonstrated significantly better swallowing impairment scores at 3 months postoperatively, the comparable adhesion scores between groups indicate that a direct anti-adhesion effect was not demonstrated in this study. To our knowledge, this is the first clinical study investigating the application of ADM specifically in anterior cervical spine surgery.

Dysphagia after ACDF surgery remains a clinically significant problem despite its high incidence [2,6]. The etiology is multifactorial and includes direct trauma to pharyngeal and esophageal structures during retraction, postoperative soft tissue swelling, adhesion formation, and hardware prominence [7,8,26]. Previous studies have suggested various risk factors with inconsistent findings across different studies [9,10,11,27].

The mechanism by which ADM may improve swallowing function despite similar adhesion scores remains unclear. ADM is a tissue-engineered scaffold that retains natural biological components, providing a biomimetic surface for cell attachment. The three-dimensional collagen matrix allows for revascularization and cell repopulation, facilitating normal tissue remodeling while minimizing inflammation. The decellularization process removes viable cells and antigens, which may contribute to its non-immunogenic properties [17,18,19,20,23]. ADM may improve swallowing function through mechanisms other than adhesion prevention, such as enhanced tissue integration or reduced local inflammation during the early healing phase.

ADM provides several desirable features as a tissue matrix: a biomimetic surface for cell attachment that matches the elasticity and stiffness of dermis; regenerative properties through modulation of mechanotransduction; durability as a matrix that persists until cellular infiltration is adequate; and non-immunogenic characteristics with degradation without producing inflammatory responses [21,22,23,28]. ADM undergoes gradual remodeling and incorporation into host tissues over time. The collagen matrix serves as a scaffold for cellular infiltration and is progressively replaced by native tissue through a process of constructive remodeling rather than encapsulation or foreign body reaction. Based on experience in other surgical applications, this process typically occurs over 3–12 months. These properties have made ADM successful in various surgical reconstructive procedures over the past two decades [29,30].

The significantly improved swallowing impairment scores in the ADM group at 3 months postoperatively suggest that ADM may provide better tissue integration compared to conventional gel-type agents. The lack of difference at immediate postoperative time points (day 1 and 3) may indicate that the beneficial effects of ADM manifest after initial tissue remodeling begins, consistent with the proposed mechanism of action involving cellular repopulation and tissue regeneration.

The comparable total adhesion scores between groups may be attributed to the questionnaire-based assessment method, which primarily evaluates subjective symptoms and clinical appearance rather than actual tissue adhesion [25]. Alternatively, the relationship between tissue adhesion and swallowing function may be more complex than previously assumed. Future studies utilizing objective imaging modalities or intraoperative assessment in revision cases may provide more accurate evaluation of anti-adhesion effects.

The absence of significant differences in perioperative inflammatory markers between groups suggests that ADM application does not induce additional systemic inflammatory response compared to conventional gel-type agents. This finding is consistent with the established non-immunogenic properties of processed ADM [19,20].

This study has several limitations. First, the SIS-6 was originally developed and validated for thyroidectomy patients, and its psychometric properties have not been formally established in the ACDF population. Additionally, a Korean-translated version was used without formal psychometric validation. However, we selected this instrument because both procedures share similar anatomical approaches involving the anterior cervical region and esophageal retraction.

Second, a significant limitation is the absence of preoperative SIS-6 baseline measurements. Although patients with pre-existing dysphagia were excluded and propensity score matching was performed, the lack of preoperative swallowing assessment limits definitive attribution of postoperative differences to the intervention.

Third, group allocation was based on period of surgery and product availability rather than randomization, which may introduce temporal confounding despite propensity matching. The propensity score model included only age, sex, and operative level. Although post hoc analysis demonstrated balance across other variables, residual confounding from unmeasured variables such as pre-existing subclinical dysphagia, esophageal retraction time, and plate profile cannot be excluded.

Fourth, although no established MCID exists specifically for SIS-6 in ACDF populations, a change of ≥2 points has been suggested as clinically meaningful in thyroidectomy literature. The observed between-group difference at 3 months (Δ = 0.42) is below this threshold, and thus the clinical significance of this statistically significant finding requires further investigation.

Fifth, the adhesion score questionnaire was originally developed for thyroidectomy. The non-significant findings may reflect either true equivalence or instrument inadequacy for the ACDF population. Individual item scores are reported in Supplementary Table S3.

Finally, the retrospective single-center design, non-randomized allocation, and relatively small sample size limit generalizability. This study may be underpowered to detect clinically meaningful differences in secondary outcomes. The follow-up period was relatively short (mean 8.8 months), and long-term safety beyond this period remains unclear. While ADM has demonstrated long-term safety in other surgical applications, its long-term behavior specifically in the anterior cervical spine has not been established. Despite these limitations, this study represents the first clinical evaluation of ADM use in anterior cervical spine surgery.

## 5. Conclusions

ACDF using novel reprocessed micronized ADM appeared safe within the follow-up period of this study and was associated with significantly improved postoperative swallowing function at 3 months compared with conventional gel-type anti-adhesive agents. However, a direct anti-adhesion effect was not demonstrated, as adhesion scores were comparable between groups. The mechanism by which ADM may improve swallowing function warrants further investigation. The clinical significance of observed differences requires further investigation given the absence of established MCID for SIS-6 in ACDF populations and the lack of preoperative baseline measurements. Larger prospective, randomized controlled studies with validated ACDF-specific outcome instruments and preoperative baseline assessment are warranted to confirm these findings and further define the role of ADM in anterior cervical spine surgery.

## Figures and Tables

**Table 1:** Baseline Demographics and Operative Data.

Variable.	ADM Group (*n* = 54)	Non-ADM Group (*n* = 54)	*p*-Value
Demographics			
Gender (Male/Female)	30 (55.6%)/24 (44.4%)	30 (55.6%)/24 (44.4%)	1.000
Age (years)	55.4 ± 11.9	55.4 ± 11.9	-
Body Mass Index (kg/m^2^)	26.7 ± 4.7	25.4 ± 5.1	0.156
Symptom Duration (months)	12.9 ± 9.1	13.7 ± 8.2	0.624
Comorbidities			
Hypertension	36 (66.7%)	36 (66.7%)	1.000
Diabetes Mellitus	26 (48.1%)	18 (33.3%)	0.118
Osteoporosis	12 (22.2%)	12 (22.2%)	1.000
Current Smoker	9 (16.7%)	6 (11.1%)	0.401
Preoperative Symptoms			
VAS Neck Pain	7.9 ± 1.0	7.6 ± 1.5	0.212
VAS Arm Pain	8.8 ± 2.1	8.7 ± 2.2	0.804
Neck Disability Index	28.7 ± 5.5	27.1 ± 9.7	0.291
Operative Data			
Operative Level			
C3–4	2 (3.7%)	2 (3.7%)	-
C4–5	11 (20.4%)	10 (18.5%)	-
C5–6	37 (68.5%)	38 (70.4%)	-
C6–7	4 (7.4%)	4 (7.4%)	0.987
Operating Time (min)	59.8 ± 8.2	59.4 ± 9.7	0.814
Blood Loss (mL) †	50.0 (35.0–75.0)	52.5 (30.0–80.0)	0.847
Hospital Stay (days)	4.2 ± 1.4	4.7 ± 1.2	0.051
Follow-up Duration (months)	8.8 ± 3.8	7.8 ± 3.2	0.140

Values are presented as mean ± SD, *n* (%), or † median (interquartile range). Continuous variables compared using independent *t*-test or Wilcoxon rank-sum test (†). Categorical variables compared using chi-square test or Fisher’s exact test.

**Table 2:** Clinical outcomes over time between ADM and non-ADM groups.

Outcome	Time Point	ADM	non-ADM	*p*-Value
VAS neck pain	Pre Op	7.9 ± 1.0	7.6 ± 1.5	0.71
	Post-1D	3.1 ± 1.4	2.8 ± 1.5	0.33
	Post-1M	2.3 ± 1.3	2.1 ± 1.4	0.41
	Post-3M	2.0 ± 1.2	1.8 ± 1.3	0.45
VAS arm pain	Pre Op	8.8 ± 2.1	8.7 ± 2.2	0.32
	Post-1D	2.4 ± 1.5	2.3 ± 1.4	0.68
	Post-1M	1.6 ± 1.2	1.9 ± 1.3	0.29
	Post-3M	1.8 ± 1.3	1.7 ± 1.2	0.74
NDI	Pre Op	28.7 ± 5.5	27.1 ± 9.7	0.69
	Post-1D	23.5 ± 6.0	21.8 ± 6.5	0.23
	Post-1M	17.8 ± 5.5	19.6 ± 6.0	0.18
	Post-3M	15.9 ± 5.0	17.8 ± 5.5	0.15

ADM, acellular dermal matrix; VAS, visual analogue scale; NDI, neck disability index; D, day; M, month. Data are presented as mean ± standard deviation.

**Table 3:** Satisfactory results by modified MacNab criteria.

Group (*n*/%)	Excellent	Good	Fair	Poor	*p*-Value
ADM	30 (55.6)	21 (38.9)	3 (5.6)	0 (-)	0.43
Non-ADM	33 (61.1)	15 (27.8)	6 (11.1)	0 (-)	

ADM, acellular dermal matrix.

**Table 4:** Postoperative changes in SIS-6 scores and prevertebral soft tissue swelling.

Parameters	Post 1D	Post 3D	Post 1M	Post 3M
SIS-6				
ADM	2.49 ± 1.41	1.75 ± 0.59	1.09 ± 0.58	0.26 ± 0.16
Non-ADM	2.67 ± 1.44	1.93 ± 0.56	1.76 ± 0.53	0.68 ± 0.27
*p*-Value	0.60	0.20	0.03 *	0.01 *
Δ Prevertebral swelling				
ADM	5.90 ± 4.16	10.53 ± 5.95	1.90 ± 0.80	1.97 ± 0.32
Non-ADM	6.70 ± 4.46	9.37 ± 5.64	2.60 ± 1.30	1.60 ± 0.77
*p*-Value	0.11	0.68	0.36	0.15

* Statistically significant (*p* < 0.05). SIS-6, Swallowing Impairment Index; ADM, acellular dermal matrix; D, day; M, month. Data are presented as mean ± standard deviation.

**Table 5:** Total Adhesion Score by Postoperative Time Point.

Time Point	ADM Group (*n* = 54)		Non-ADM Group (*n* = 54)		Between-Group *p*-Value ‡
	Score	*p* vs. PreOp †	Score	*p* vs. PreOp †	
Preoperative	8.72 ± 4.01	—	8.92 ± 4.14	—	0.895
Postoperative	13.20 ± 7.78	<0.001	13.84 ± 7.48	<0.001	0.162
Post-1 Month	11.37 ± 5.13	<0.001	11.79 ± 5.83	<0.001	0.652
Post-3 Months	9.69 ± 4.23	<0.001	9.88 ± 3.83	<0.001	0.727

Values are presented as mean ± SD. Total adhesion score ranges from 0 (no symptoms) to 28 (maximum symptoms). † Within-group comparison: *p*-value for change from preoperative baseline within each group (paired *t*-test). ‡ Between-group comparison: *p*-value comparing ADM vs. Non-ADM groups at each time point (independent *t*-test).

**Table 6:** Inter- and intra-observer reliability of radiological measurements.

Time Point	Inter-Observer ICC (95% CI)	Intra-Observer ICC (95% CI)
Post 1D	0.91 (0.86–0.95)	0.88 (0.82–0.93)
Post 3D	0.93 (0.89–0.96)	0.90 (0.85–0.94)
Post 1M	0.94 (0.90–0.97)	0.92 (0.88–0.95)
Post 3M	0.92 (0.87–0.96)	0.89 (0.83–0.94)

ICC, intraclass correlation coefficient; CI, confidence interval; D, day; M, month.

**Table 7:** Perioperative laboratory parameters.

Parameter	Time Point	ADM	Non-ADM	*p*-Value
CK (U/L)	Pre Op	71.8 ± 23.6	67.2 ± 22.9	0.43
	Post-1D	168.5 ± 52.7	154.3 ± 48.9	0.31
	Post-2W	59.1 ± 21.8	62.4 ± 22.1	0.54
LDH (U/L)	Pre Op	258.4 ± 61.9	269.6 ± 64.2	0.40
	Post-1D	382.4 ± 92.1	358.7 ± 89.6	0.28
	Post-2W	204.7 ± 63.2	216.3 ± 65.4	0.45
CRP (mg/dL)	Pre Op	0.52 ± 0.43	0.61 ± 0.46	0.35
	Post-1D	2.84 ± 1.36	3.12 ± 1.48	0.27
	Post-2W	1.08 ± 0.62	1.26 ± 0.71	0.30
ESR (mm/hr)	Pre Op	12.1 ± 7.8	17.4 ± 9.2	0.24
	Post-1D	34.8 ± 12.6	31.9 ± 11.8	0.34
	Post-2W	19.7 ± 9.8	15.4 ± 8.9	0.22

CK, creatine kinase; LDH, lactate dehydrogenase; CRP, C-reactive protein; ESR, erythrocyte sedimentation rate; ADM, acellular dermal matrix; D, day; W, week. Values are presented as mean ± standard deviation.

## Data Availability

The data presented in this study are available on request from the corresponding author.

## Supplementary Materials

The following supporting information can be downloaded at: https://www.mdpi.com/article/10.3390/medicina62061163/s1, Supplementary Table S1: Swallowing Impairment Score (SIS-6) Questionnaire; Supplementary Table S2: Standardized Mean Differences Before and After Propensity Score Matching; Supplementary Table S3: Individual Adhesion Score Item Results at 3 Months; Supplementary Checklist S1: STROBE Statement Checklist for Cohort Studies.
