# Supplementary material for: Effect of Reprocessed Micronized Acellular Dermal Matrix on Postoperative Dysphagia After Anterior Cervical Discectomy and Fusion: A Propensity Score-Matched Study
Source: Medicina (Kaunas). 2026 Jun 15;62(6):1163. doi: 10.3390/medicina62061163 (PMC13304184; doi:10.3390/medicina62061163)
Supplement: Supplementary file 1 [file medicina-62-01163-s001.zip › medicina-4294867-supplementary.pdf]

Supplementary Materials

Effect of Reprocessed Micronized Acellular Dermal Matrix on Postoperative Dysphagia After Anterior Cervical Discectomy and Fusion: A Propensity Score-Matched Study

Supplementary Table S1. Swallowing Impairment Score (SIS-6) Questionnaire.

The SIS-6 is a validated 6-item self-evaluation questionnaire originally developed by Lombardi et al. (2006) for assessing swallowing dysfunction following thyroidectomy. Each item is scored from 0 to 4, with a total score ranging from 0 (no impairment) to 24 (maximum impairment).

Scoring: 0 = Never; 1 = A little; 2 = Sometimes; 3 = Often; 4 = Always

Item	Question
1	Do you have difficulty swallowing solid foods?
2	Do you have difficulty swallowing liquids?
3	Do you have difficulty swallowing pills or tablets?
4	Do you feel a sensation of food sticking in your throat?
5	Do you experience coughing or choking when swallowing?
6	Do you have pain when swallowing?

Reference: Lombardi CP, Raffaelli M, D'Alatri L, et al. Voice and swallowing changes after thyroidectomy in patients without inferior laryngeal nerve injuries. Surgery. 2006;140(6):1026–1032.

Supplementary Table S2. Standardized Mean Differences (SMD) Before and After Propensity Score Matching.

Variable	SMD Before Matching	SMD After Matching
Age (years)	0.18	0.02
Sex (male)	0.22	0.00
BMI (kg/m²)	0.15	0.05
Smoking status	0.12	0.04
Diabetes mellitus	0.08	0.03
Hypertension	0.10	0.06
Symptom duration (months)	0.14	0.07
Operative level (C5-6)	0.25	0.04
Operative time (minutes)	0.11	0.08
JOA score	0.09	0.05
Preoperative VAS neck	0.13	0.06
Preoperative VAS arm	0.16	0.04
Preoperative NDI	0.14	0.07

SMD = Standardized Mean Difference. Values < 0.1 indicate adequate balance between groups.

Note: Propensity scores were calculated using logistic regression with age, sex, and operative level as covariates. One-to-one nearest-neighbor matching was performed with a caliper width of 0.2 standard deviations of the logit of the propensity score.

Supplementary Table S3. Individual Adhesion Score Item Results at 3 Months.

Item	ADM Group	Non-ADM	p-value
Cervical appearance	0.8 ± 0.4	0.9 ± 0.5	0.32
Symmetry/comfort	0.7 ± 0.5	0.8 ± 0.4	0.41
Swallowing saliva	0.3 ± 0.3	0.4 ± 0.4	0.28
Swallowing water	0.2 ± 0.2	0.3 ± 0.3	0.18
Swallowing solid food	0.4 ± 0.3	0.6 ± 0.4	0.09
Neck tightness/stiffness	0.6 ± 0.4	0.7 ± 0.5	0.35
Total score	3.0 ± 1.2	3.7 ± 1.4	0.12

Values are presented as mean ± SD. Each item is scored 0–4 (0 = no impairment, 4 = maximum impairment). The adhesion score questionnaire was originally developed for thyroidectomy (Kim JK et al. 2022) and has not been validated for ACDF populations.

Supplementary Checklist S1. STROBE Statement Checklist for Cohort Studies.

Item No.	Recommendation	Reported on Page/Section	Compliant
1a	Title: Indicate study design	Title	Yes
1b	Abstract: Provide informative summary	Abstract	Yes
2	Background/rationale	Introduction	Yes
3	Objectives	Introduction	Yes
4	Study design	Methods 2.1	Yes
5	Setting	Methods 2.1	Yes
6a	Eligibility criteria	Methods 2.2	Yes
6b	Sources and methods of selection	Methods 2.2	Yes
7	Variables defined	Methods 2.3, 2.4	Yes
8	Data sources/measurement	Methods 2.3	Yes
9	Bias	Methods 2.4	Yes
10	Study size	Methods 2.4	Yes
11	Quantitative variables	Methods 2.4	Yes
12a	Statistical methods	Methods 2.4	Yes
12b	Subgroups and interactions	N/A	N/A
12c	Missing data	Results 3.1	Yes
12d	Matching methods	Methods 2.4	Yes
12e	Sensitivity analyses	N/A	N/A
13a	Participants: Numbers	Results 3.1	Yes
13b	Reasons for non-participation	Results 3.1	Yes
13c	Flow diagram	N/A	No
14a	Descriptive data	Table 1	Yes
14b	Follow-up time	Results 3.1	Yes
15	Outcome data	Tables 2-7	Yes
16a	Unadjusted estimates	Results 3.2-3.6	Yes
16b	Category boundaries	Methods 2.3	Yes
16c	Relative vs absolute risk	N/A	N/A
17	Other analyses	Results 3.3	Yes
18	Key results	Discussion	Yes
19	Limitations	Discussion	Yes

20	Interpretation	Discussion	Yes
21	Generalizability	Discussion	Yes
22	Funding	Funding statement	Yes

Reference: von Elm E, Altman DG, Egger M, et al. The Strengthening the Reporting of Observational Studies in Epidemiology (STROBE) statement: guidelines for reporting observational studies. Lancet. 2007;370(9596):1453–1457.
